# Comprehensive evaluation of Reseda lutea L. (Wild Mignonette) and 7 isolated flavonol glycosides: determination of antioxidant activity, anti-Alzheimer, antidiabetic and cytotoxic effects with in vitro and in silico methods

**DOI:** 10.55730/1300-0527.3426

**Published:** 2022-04-08

**Authors:** Hatice KIZILTAŞ

**Affiliations:** Department of Pharmacy Services, Van Vocational School of Health Services, Van Yüzüncü Yıl University Van, Turkey

**Keywords:** Antioxidant activity, cytotoxicity, enzyme inhibition, molecular docking, *Reseda lutea*

## Abstract

In this study, anticholinergic, antidiabetic, antioxidant and cytotoxic activities of *Reseda lutea* L. (*R. lutea*) were determined. Ethanol extracts of *R. lutea* (EERL) and water extract of *R. lutea* (WERL) were prepared for biochemical analysis. The antioxidant capacities of EERL and WERL were evaluated with 6 different methods. In addition, acetylcholinesterase (AChE), α-amylase and *α-*glycosidase enzyme inhibition by EERL were measured. According to the results, EERL exhibited high inhibition effects against α-amylase, α-glycosidase and AChE enzymes. The IC_50_ values of EERL against AChE (2.21 μg/mL), α-glycosidase (1.38 μg/mL), and α-amylase (0.11 μg/mL) were determined. Also, high cytotoxic effect of EERL was observed on human lung cancer cell lines (A549) with an *IC*_50_ value of 3.58 ± 1.10 μg/mL. The affinities of 7 kaempferol and isorhamnetin rhamnopyranoside molecules, previously isolated from *R. lutea*, for AChE, α-amylase and, α-glycosidase were determined by molecular docking studies. Molecular docking results supported the in vitro results of the study. The results showed that the aerial parts of *R. lutea* have effective antioxidant, anticholinergic, antidiabetic and cytotoxic activities. This research will form the basis for further studies about *R. lutea* usage for drug development.

## 1. Introduction

**Figure 1:**
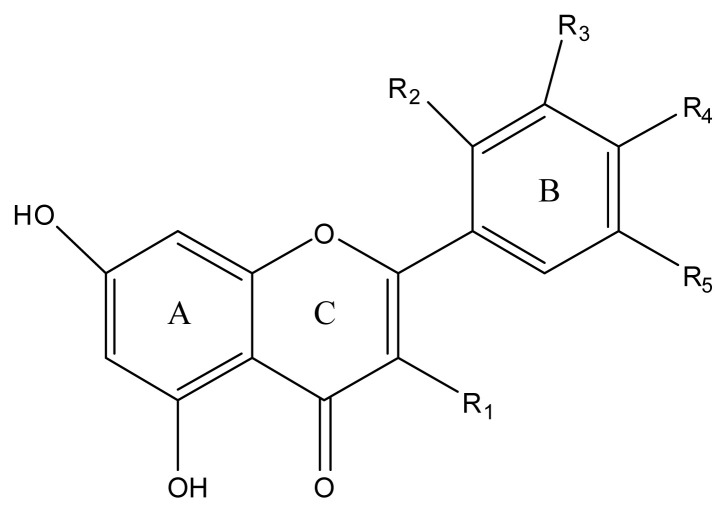
Basic chemical structure of flavonoids.

Antioxidants are substances which neutralize free radicals and prevent damage from oxidative stress caused by an increase in free radicals and therefore are vital particles [1]which address the mechanism of antioxidant activity and focus on the kinetics of the reactions including the antioxidants. Many studies evaluating the antioxidant activity of various samples of research interest using different methods in food and human health have been conducted. These methods are classified, described, and discussed in this review. Methods based on inhibited autoxidation are the most suited for termination-enhancing antioxidants and for chain-breaking antioxidants, while different specific studies are needed for preventive antioxidants. For this purpose, the most common methods used in vitro determination of antioxidant capacity of food constituents were examined. Also, a selection of chemical testing methods was critically reviewed and highlighted. In addition, their advantages, disadvantages, limitations and usefulness were discussed and investigated for pure molecules and raw extracts. The effect and influence of the reaction medium on the performance of antioxidants are also addressed. Hence, this overview provides a basis and rationale for developing standardized antioxidant methods for the food, nutraceuticals, and dietary supplement industries. In addition, the most important advantages and shortcomings of each method were detected and highlighted. The chemical principles of these methods are outlined and critically discussed. The chemical principles of methods of 2,2′-azinobis-(3-ethylbenzothiazoline-6-sulphonate. Oxidative stress can cause cellular damage such as lipid peroxidation, DNA and protein damage, and ultimately lead to diseases such as cardiovascular disease, inflammation, atherosclerosis, diabetes, and cancer [2]. Antioxidants, on the other hand, can inhibit or reduce the effect of oxidative stress on lipids, proteins and DNA due to their ability to scavenge free radicals [3]. Dietary antioxidants play an important function in reducing oxidative stress by helping endogenous antioxidants [4]. Phytonutrients that prevent free radical damage like vitamin C, tocopherols, flavonoids, carotenoids and phenolic acids are sources of potential antioxidants [5]. Various synthetic antioxidants are currently widely used; however, there have been suspicions that these compounds have toxic and carcinogenic effects. Thus, recently interest has increased significantly in the discovery of natural antioxidants to replace synthetic antioxidants that are restricted by their carcinogenicity [6]. Almost 50% of all the prescription drugs used in cancer therapy are natural products or are obtained directly from natural products. In other fields, natural compounds are used even more; for example, to develop drugs for new infectious diseases [7]. Still most of the world’s population is dependent on nearly 250,000 plant species as the main drug sources [8]. A nutritious diet rich in antioxidant compounds is important for health [9]. Dietary antioxidants are effective in preventing many neurodegenerative disorders, cardiovascular diseases, cancer, Parkinson’s and Alzheimer’s diseases (AD) [10]. All of these have led to an increasing trend in consumer preferences towards safer and natural antioxidants, thereby accelerating the discovery of natural antioxidants [5]. Many phytochemicals, such as phenols and polyphenols, are the main components of plants, which display antioxidant activity to protect against oxidative reactions [1]. They contain metal chelators, radical chain reaction inhibitors, antioxidant enzyme cofactors and inhibitors of oxidative enzymes in order to scavenge ROS/RNS to stop radical chain reactions or their first occurrence [11]. Therefore, plants which contain phenolic compounds and flavonoids are beneficial for human health due to their antioxidant, antiallergic, anticancer, antiviral and antimicrobial properties. They are also known to be potent inhibitors of many enzymes, such as cyclooxygenase, xanthine oxidase, lipoxygenase, and phosphoinositide 3-kinase [8,12]. Flavonoids can induce apoptosis through some modulations of the apoptosis-related cellular signal transduction pathway. Some studies showed that flavonoids can exert regulatory activities in cells through actions in different signal transduction pathways such as kinases, caspases, and Bcl-2 family members [13]. The basic flavonoid structure is the flavan nucleus, which consists of 15 carbon atoms arranged in three rings (C6–C3–C6) (diphenylpropane) (Figure 1) which are labeled A, B, and C [1]. Studies of the glycosylation of flavonoid aglycone-based compounds have great general interest due to obtaining novel compounds with high stereo- and regioselectivity [14].

The Resedacea family is represented by six genera and 75 species, which are distributed predominantly in the Mediterranean region and in South West Asia region [15]. *Reseda lutea* L. (*R. lutea*) is linked to the Resedaceae family, and 14 species of *R. lutea* are found in Turkey, seven of them endemic [16]. *R. lutea* is known as yellow mignonette or wild mignonette, and is used as a medicinal plant [17]. Many studies demonstrated the existence of flavonoids, anthocyanins, and α-glycosides in the aerial parts of *R. lutea* [18–20]. *R. lutea* was included in lists of interesting species at the ethnopharmacological level because of its healing properties, antitumor, anti-HIV, cytotoxic, antibacterial, antiinflammatory, and antioxidant effects [7,16,21–23]. Kaempferol isolated from *R. lutea* is a flavonoid antioxidant found in fruits and vegetables. In many studies, it was stated that kaempferol has positive effects against chronic disease, especially cancer and nervous system diseases [24].

**Figure 2:**
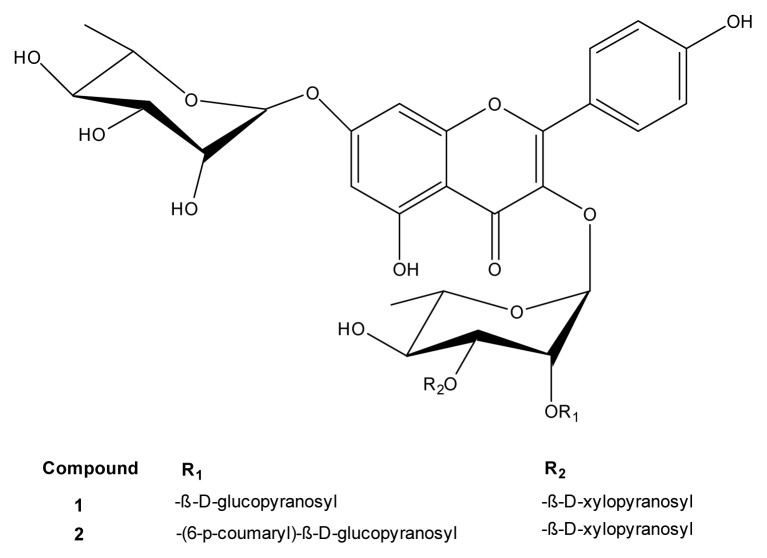
Chemical structure of isolated compounds 1–2 isolated from aerial parts of *R. lutea*.

**Figure 3:**
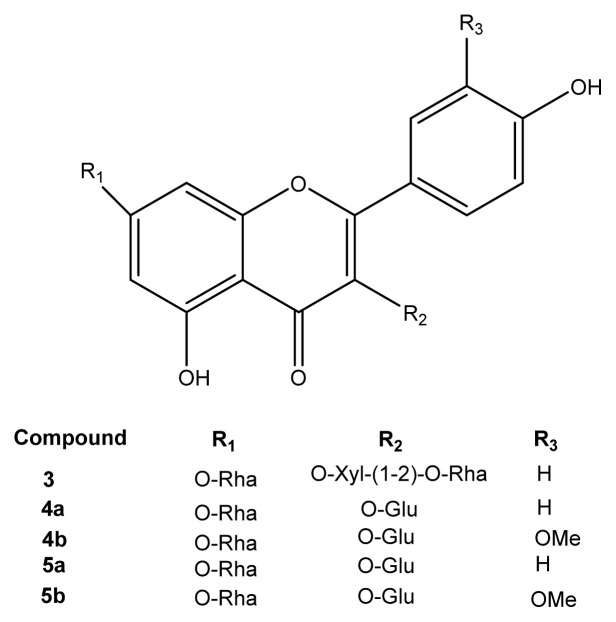
Chemical structure of isolated compounds 3–5b isolated from aerial parts of *R. lutea*.

Our research team isolated many flavonoids from the aerial parts of *R. lutea*. The isolation and structure determination of two new (1–2) kaempferol rhamnopyranosides and five known (3–7) kaempferol and isorhamnetin rhamnopyranosides (Figures 2 and 3) from *R. lutea* in our previous research [20]. Here, the molecular docking of these isolated flavonoid compounds with AChE, α-glycosidase and α-amylase are described, along with *in vitro* antioxidant activity, anti-Alzheimer and antidiabetic enzyme inhibition and cytotoxic activities of *R. lutea* extracts.

## 2. Materials and methods

### 2.1. Chemicals

Compounds which are used for antioxidant activity suchlike neocuproine (2,9-dimethyl-1,10-phenanthroline), 2,2-azino-bis3-ethylbenzthiazoline-6-sulfonic acid (ABTS), 1,1-diphenyl-2-picryl-hydrazyl (DPPH), 3-(2-pyridyl)-5,6-bis (4-phenyl-sulfonic acid)-1,2,4-triazine (Ferrozine), ascorbic acid, BHT (butylated hydroxytoluene), α-tocopherol were obtained from Sigma (Sigma-Aldrich GmbH, Steinheim, Germany). All other chemicals used were of analytical grade and obtained from either Sigma-Aldrich or Merck Millipore.

### 2.2. Identification and collection of the plant material

The aerial parts of *Reseda lutea* L. were collected from Evyapan, Kağızman, Kars in July 2019 (location: 40°02′36.2″N 43°02′42.6″E, herbarium code: M. Pınar 16412). The samples were authenticated by Assoc. Prof. Dr. S. Mesut PINAR (Van YYU, Faculty of Sciences, Department of Biology) and voucher specimens were deposited in the Herbarium of the Biology Department (VANF), Van, Turkey.

### 2.3. Isolated compounds

The kaempferol and isorhamnetin rhamnopyranosides were isolated from the aerial parts of *R. lutea*. Isolation of the seven kaempferol and isorhamnetin rhamnopyranoside compounds was previously reported by [20] and their structures are shown in Figures 1 and 2. As a result of the study two new compounds isolated and identified for the first time were named as **1** and **2**. Of the seven isolated and identified compounds, the remaining five were previously known molecules and named as (**3–7**). In this study, molecules were named with the same numbers so that they can be easily distinguished. Two new compounds named kaempferol-3-O-[2-O-(β-D-xylopyranosyl)-3-O-(β-D-glucopyranosyl)]-α-L-rhamnopyranosyl-7-O-α-Lrhamnopyranoside (**1**) and kaempferol-3-O-[2-O-((6-O-trans-p-coumaryl)-β-Dglucopyranosyl)-3-O-(β-D-xylopyranosyl)]-α-L-rhamnopyranosyl-7-O-α-L-rhamnopyranoside (**2**) are reported, as the first tetrasaccharidic secondary metabolites from the family Resedaceae [20]. The known compounds kaempferol-3-O-[2-O-(β-D-xylopyranosyl)]-α-L-rhamnopyranosyl-7-O-α-L rhamnopyranoside (**3**); kaempferol-3-Oβ-D-glucopyranosyl-7-O-α-L-rhamnopyranoside (**4a**) and isorhamnetin-3-O-β-D-glucopyranosyl-7-O-α-L-rhamnopyranoside (**4b**), 4a (major) and 4b (minor) were isolated as a mixture; kaempferol-3,7di-O-α-L-rhamnopyranoside (**5a**) and isorhamnetin-3,7-di-O-α-rhamnopyranoside (**5b**), 5a (major) and 5b (minor) were isolated as a mixture and identified by comparison with their IF-I-NMR spectra. The data are stated in our previous publication [20].

### 2.4. Preparation of ethanol and water extracts

The procedure for the extractions was applied as described previously [25,26]. To prepare evaporated ethanolic (EERL) and lyophilized water extracts of aerial parts of *R. lutea* (WERL), 25 g of air-dried plant material was finely pulverized for each extract in a grinder. The aquatic extract was prepared by boiling with 0.5 L water then the sample was lyophilized in a lyophilizer (Labconco, Freezone 1L) at −50° C with 5 mm-Hg= pressure setting [25]. The ethanolic extract was prepared by soaking in 0.5 L ethanol, then the solvent was evaporated via a rotary evaporator (Heidolph Hei-VAP HL, Germany) [27]. Both lyophilized and evaporated extracts were stored at −20°C until their use.

### 2.5. Antioxidant activity

#### 2.5.1. Reducing ability assay

*R. lutea*’s reducing ability was carry out by three separate methods which are; the Fe^3+^ reducing ability [27], copper ions (Cu^2+^) reduction capacity (CUPRAC) [28] and ferric reducing antioxidant power (FRAP) methods [12].

#### 2.5.2. Radical scavenging methods

DPPH^•^ scavenging [29], Fe^2+^ chelating [30,31] and ABTS^+•^ scavenging activities [32] are 3 separate methods which are based on spectrophotometric measurements. They all were used for the determination of free radical scavenging potential of WERL and EERL.

#### 2.5.3. Total phenolics and flavonoids contents

The total amount of phenolics in WERL and EERL were determined from the method in previous research. Gallic acid was used as standard and the amount of the total phenolic content in samples was determined as micrograms of gallic acid equivalents (GAE) [25]. The total amount of flavonoids found in WERL and EERL was determined by the method described in a previous study [26]. The quantity was determined in micrograms of quercetin equivalents (QE) using the equation obtained from the standard quercetin plot.

### 2.6. Enzyme inhibitory activities

The AChE enzyme inhibitory properties of EERL were measured according to a previous study, using electric eel as source of the AChE enzyme [33]. The α-amylase and α-glycosidase inhibitory effects of EERL were estimated according to a method from previous studies [34]. The IC_50_ value is defined as the concentration of compound causing 50% inhibition and was obtained from activity (%) against compounds graphs [35].

### 2.7. Molecular docking studies

The chemical structures of the isolated compounds were drawn with ChemDraw (CambridgeSoft, USA) and optimized using Chem3D version. The chemical structure of α-glycosidase (PDB ID: 3A4A), α-amylase (PDB ID: 3L2M) and acetylcholinesterase (PDB ID: 1ACJ) enzymes were downloaded from the “Protein Data Bank” website. The structures of these enzymes were optimized in AutoDock-Tools 1.5.7 [36]. Structure optimization and the most stable conformations of the ligands were determined with AutoDockTools then the PDBQT file of the ligands was prepared. The optimized enzyme and ligand structures were loaded into AutoDock-Tools and the same program was used for docking. The best docking energy scores and binding interactions were analyzed with PLIP [37]and BIOVIA Discovery Studio.

### 2.8. Cytotoxic activity

#### 2.8.1. Cell culture

Commercially available human respiratory epithelial cell line (A549) (human lung cancer cell lines) was used in the study. Cells were cultured in appropriate medium (90% medium + 10% serum + 1% antibiotic) in 25 cm2 flasks (BINDER CB 150 E3, Germany) in the incubator (37±1 °C containing 5% CO2, approximately 95% relative humidity).

#### 2.8.2. Cell viability assay (MTT)

After seeding with in a 96-well plate, cells were allowed to incubate for 24 h to adhere to the bottom of the plate. After 24 h, solutions with varying concentrations (0.0064–100 μg/mL) prepared from the ethanolic extract of the aerial parts of *R. lutea* (EERL) were added to the cells. After 24 h incubation, 20 μL of stock MTT solution (filtered MTT solution dissolved in 5 mg/mL PBS) was added to each well, the plate was left in the dark, and incubated for 3 h. The medium of the cells was completely withdrawn and 100 μL of DMSO was added. The plate was shaken in the dark for 15 min (orbital shaker, Heidolph Unimax 1010, Germany) and after the formazan crystals were dissolved, they were read in a multifunctional plate reader (BioTek Synergy HTX, USA) at a wavelength of 540 nm.

### 2.9. Statistical analysis

All extractions and analyses were performed in triplicate with five replicates for the MTT test, and data are presented as mean ± SD values. One-way analysis of variance (ANOVA) test was used. Differences between groups were examined with Duncan and Tukey correction. Statistical significance levels were taken as p < 0.05 significant, and p < 0.001 highly significant. SPSS statistical software version 25.0 package was used for analysis.

## 3. Result and discussion Antioxidant activity

In this study, the antioxidant potential of EERL and WERL was measured by various in vitro spectrophotometric techniques, including DPPH^•^ and ABTS^+•^ scavenging, Fe^2+^ chelating, Fe^3+^ reduction, CUPRAC and FRAP reduction assays. The antioxidant profile of ethanol and water extracts from the aerial parts of *R. lutea* are shown in Table 1 and ure 4, as characterized using ferric ion (Fe^3+^) reduction, cupric ion (Cu^2+^) reducing capacity (CUPRAC) and Fe^3+^-TPTZ reducing (FRAP) assays.

**Figure 4:**
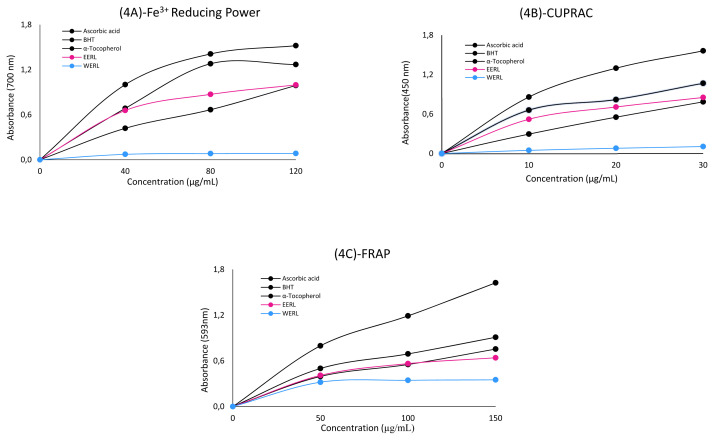
The reducing abilities of the WERL, EERL and standard antioxidant compounds.

Reduction activity tests are very important to determine the antioxidant capacity of a molecule. The first method used was to reduce Fe^3+^ to Fe^2+^ in Fe[(CN)_6_]^3+^ solution, which is one of the important methods [1]. Reducing power of EERL, WERL and positive controls increased steadily with increasing concentration in the samples (40–120 μg/mL) and were as follows: ascorbic acid (1.52 ± 0.03, r^2^: 1.00) > BHT (1.27 ± 0.01, r^2^: 0.99) > EERL (1.00 ± 0.01, r^2^: 0.99) > α-tocopherol (0.99 ± 0.01, r^2^: 0.99) > WERL (0.08 ± 0.01, r^2^: 0.97) (Table 1 and Figure 4A). According to the results, *R. lutea* has the ability to reduce ferric ions (Fe^3+^) to a significant level to remove free radicals and has electron donating properties. The reducing power of EERL was higher than α-tocopherol and the standard antioxidant used (p < 0.001) (Table 1), but it was lower than ascorbic acid and BHT. The result of the reduction reaction, radical chain reactions which can be quite damaging, was terminated [1].

Reducing power of positive controls, EERL and WERL (30 μg/mL) for cupric ions (Cu^2+^) (CUPRAC) is demonstrated in Table 1 and Figure 4B. The cupric ion (Cu^2+^) reducing power was as follows: BHT (1.56 ± 0.09, r^2^: 1.00) > ascorbic acid (1.07 ± 0.01, r^2^: 0.97) > EERL (0.85 ± 0.01, r^2^: 0.99) > α-tocopherol (0.79 ± 0.07, r^2^: 1.00) > WERL (0.11± 0.01, r^2^: 1.00).

The Fe^3+^-TPTZ reduction method (FRAP) was used as the last method, which is one of the reduction forcing methods of Fe^+3^ to Fe^2+^ [38]. The reducing power of the standards and *R. lutea* decreased in the following order: ascorbic acid (1.62 ± 0.02, r^2^: 0.99) > BHT (0.91 ± 0.01, r^2^: 0.99) > α-tocopherol (0.76 ± 0.08, r^2^: 0.99) > EERL (0.64 ± 0.03, r^2^: 0.99)> WERL (0.35 ± 0.01, r^2^: 0.96) (Table 1 and Figure 4C). The high reduction capability of the Fe^3+^-TPTZ complex is displayed through high absorbance values in this method. Also, *R. lutea* extracts were determined to have an effective FRAP reduction ability (p < 0.001) (Table 1). *R. lutea* plant extracts used in all three reduction methods ensured reduction to a value close to the standards. In addition, BHT and ascorbic acid which were used as standards provided the best results.

**Figure 5:**
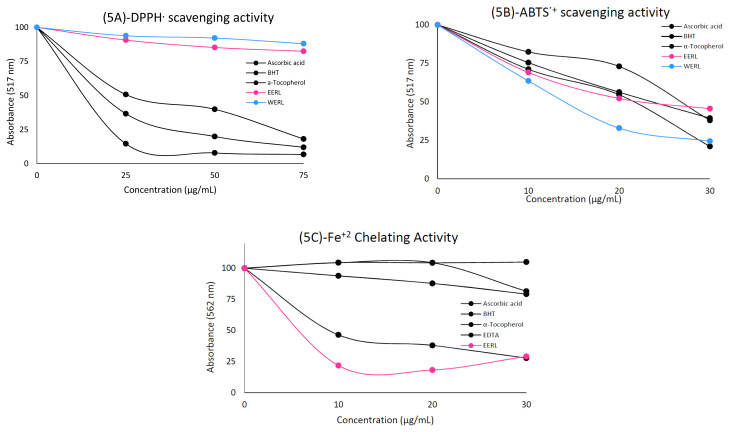
Radical scavenging activity of the EERL, WERL and standard antioxidant compounds.

According to the DPPH radical scavenging activity method, by mixing the DPPH solution with a substance capable of donating a hydrogen atom, the reduced form occurs and the purple color of the solution turns yellow, which can be observed as a decrease in absorbance [39]. Lower IC_50_ values suggests an effective scavenged DPPH^•^ scavenging effect. DPPH assay is widely used to determine the radical scavenging activity spectrophotometrically [40]. The DPPH free radical scavenging activities of ethanol and water extracts from the aerial parts of *R. lutea* and positive controls were investigated. In addition, we determined the IC_50_ values of extracts and standard antioxidant compounds. The IC_50_ values for DPPH^•^ scavenging by extracts and standard antioxidants were determined in the following order: ascorbic acid (16.12 ± 0.01, r^2^: 0.96) > α-tocopherol (23.10 ± 0.03, r^2^: 0.98) > BHT (31.50 ± 0.01, r^2^: 0.98) > EERL (231.0 ± 0.01, r^2^: 0.95) > WERL (346.50 ± 0.03, r^2^: 0.96) (Table 1 and Figure 5A) (p < 0.001). Ascorbic acid had the most effective DPPH^•^ scavenging activity. When we examined the results of previous studies, there was only one study that investigated the DPPH^•^ scavenging activity. According to this study, the IC_50_ value for the aqueous extract of *R. lutea* flowers was found to be 13.4 ± 8.6% inhibition (Table 1) [41]. When the results are evaluated together with our own results, IC_50_ values are quite high in general studies; however, the highest result was obtained in our study. This indicates that *R. lutea* extracts do not have very strong DPPH^•^ scavenging activity.

The ABTS^•^^+^ scavenging experiment is based on the inhibition of ABTS^•^^+^ antioxidants. According to the method, a stable form of the radical is produced in the experiment and reacts with an antioxidant to form blue-green ABTS^•^^+^, color removal indicates the rate of ABTS^•^^+^ inhibition [42]. Both EERL and WERL showed scavenging activity against ABTS^•^^+^. The IC_50_ values of ABTS^•^^+^ scavenging for extracts and standard antioxidants were determined in the following order: WERL (14.14 ± 0.04, r^2^: 0.99) > α-tocopherol (15.40 ± 0.01, r^2^: 0.99) > EERL (23.90 ± 0.01, r^2^: 0.97) ≈ Ascorbic acid (23.10 ± 0.01, r^2^: 1.00) > BHT (26.65 ± 0.01, r^2^: 0.97) (Table 1 and Figure 5B). In the study conducted by Kang et al. (2013), the ABTS^•^^+^ scavenging activity was 10.2 ± 0.4% inhibition for the aqueous extract of *R. lutea* flowers. When the results of our research and others are evaluated, the IC_50_ values are quite low (p < 0.001) (Table 1). These results indicate that *R. lutea* extracts have very strong ABTS^•^^+^ scavenging activity.

Metal chelating activity of *R. lutea* and standard antioxidant compounds were evaluated and IC_50_ values were determined (Table 1 and Figure 5C). EERL was determined to have the most effective chelating activity (p < 0.001) (Table 1). The IC_50_ values for the metal chelating activity of extracts and standard compounds were determined in the following order: EERL (11.18 ± 0.05, r^2^: 0.96) > BHT (14.75 ± 0.06, r^2^: 0.97) > ascorbic acid (99.0 ± 0.04, r^2^: 1.00) > EDTA (231.0 ± 0.31, r^2^: 0.95) > α-tocopherol (330.0 ± 0.02, r^2^: 0.91). The results indicate that *R. lutea* extract has very strong metal chelating activity.

Considering the six in vitro methods evaluated, in summary EERL and WERL were determined to have very high antioxidant potential when compared with the standards. EERL and WERL exhibited higher antioxidant activity values than standards in some experiments. This situation is in parallel with the high amount of phenolic and flavonoid content they contain. In four experiments out of these six in vitro methods, EERL exhibited more potent antioxidant potential than WERL, which may be attributed to the better dissolution of organic compounds and phenolic acids in ethanol.

Phenolic compounds play important roles in antioxidant activity and stabilization of lipid peroxidation [25]. Total phenolic contents were calculated using standard gallic acid calibration curves. The total phenolic contents of EERL and WERL were determined as 47.73 ± 0.32 to 7.73 ± 0.13 μg GAE, respectively (Table 2). EERL had higher amounts of phenolic compounds in both extracts. Many physiological benefits were attributed to flavonoids, including protection from cancer and cardiovascular disease, due to the powerful antioxidant and free radical scavenging properties they possess [43]. Flavonoids are known to have effects on signal transduction pathways related to cellular proliferation, differentiation, cell cycle progression, apoptosis, inflammation, angiogenesis, and metastasis [13]. The amount of total flavonoid in EERL and WERL was determined as 84.43 ± 2.72 and 9.83 ± 0.41 μg quercetin equivalent, respectively (Table 2). Considering the results, there was a positive correlation between the total flavonoid content in EERL and WERL and their antioxidant activity. In a study, the aqueous extract from the aerial parts of *R. lutea* was investigated and the total phenol content was 10.8 ± 1.4 GAE mg/g [41]. In another study conducted in 2019, the seeds of *R. lutea* were extracted with 70% aqueous ethanol and the amounts of total phenol and flavonoid were found to be 65.32 ± 3.72 mg GAE/g and 21.93 ± 2.67 mg RU/g, respectively [16]. In another study, leaves of *R. lutea* were extracted with methanol and total phenol and flavonoid were determined as 133.52 ± 0.02 mg GAE/L and 196.80 ± 0.01 mg QE/L, respectively [19]. It was observed that the results obtained from previous studies are sometimes higher and sometimes lower than our results. This is thought to be due to the differences in the ecological and soil structure of the region where the plant is grown, analysis methods, solvents and extraction conditions.

### 3.2. Enzyme inhibition

When we evaluated the results of cholinesterase enzyme inhibition, the AChE inhibition of EERL was at a lower level compared to the control (Table 3). IC_50_ values of EERL for AChE were measured as 2.21 μg/mL, while tacrine was used as positive control for AChE inhibition with a IC_50_ value of 0.124 μM against AChE [46]. A very low IC_50_ value of AChE means very high inhibition, and EERL showed an IC_50_ value was not high but efficient. AD is a chronic neurodegenerative brain disease characterized by oxidative stress, dementia, and memory impairment in the elderly, and nowadays there are few drugs with various side effects for AD [47]. This situation makes it an effective force in conducting research to define plant products to create new medicines with protective effects for the treatment of AD [48]. Modulation of acetylcholine levels at synapses by AChE inhibitors is one of the useful methods used to treat AD [49]. It has long been known that medicinal herbs can be used as a source of cholinesterase enzyme inhibitors. Medicinal plants contain phenolic compounds which give them the ability to inhibit cholinergic enzymes. Some of them were reported to acquire high AChE inhibition [9]. Kaempferol was reported to have very potent neuroprotective effect through modulation of various pathways involved in AD progression [24].

When we evaluate the results for antidiabetic enzyme inhibition of *R. lutea*, which was determined by using α-glycosidase and α-amylase enzymes, the IC_50_ values for α-glycosidase were measured as 1.38 μg/mL for EERL and 22.80 μM for acarbose [44]. In addition, the IC_50_ values for α-amylase were measured as 0.11 μg/mL for EERL and 10.01 μM for acarbose, respectively (Table 3) [45]. When the results are evaluated, EERL has higher affinity than α-amylase and α-glycosidase enzymes. Furthermore, α-glycosidase and α-amylase inhibition by EERL was more effective than acarbose, which is a standard inhibitor. Diabetes mellitus, which is a metabolic disease that causes hyperglycemia due to defects in insulin secretion and/or action, can cause chronic hyperglycemia that leads to dysfunction and failure of various organs such as nerves, eyes, heart, kidneys, and blood vessels [50]. During hyperglycemia, increased flow of glucose causes osmotic stress and cellular damage, especially in lenses [51]. It is known that these effects cause one or more of the secondary diabetic complications such as nephropathy, cataract, retinopathy, and neuropathy in patients with DM [52]. The α-glycosidase and α-amylase enzymes hydrolyze polysaccharides, converting them into simple sugar units or monosaccharides. Inhibition of the two enzymes is considered a route for therapeutic trials for DM treatment. Many plant species have high α-glycosidase and α-amylase inhibition [9]. Worldwide 800 herbs were reported to have strong antidiabetic potential, mainly by increased insulin secretion, α-glycosidase inhibitory activity, antiinflammatory effects, increased insulin resistance, regeneration of pancreatic β cells, and reduced diabetes-associated oxidative stress. Plenty of natural drugs mediate increased glucose uptake and suppression of hepatic glucose output by reducing glycogen degradation and gluconeogenesis, as well as glycolysis, glucose oxidation and glycogenesis [53]. It was determined that kaempferol improves hyperglycemia and also plays a beneficial role in diabetes by preventing oxidative damage in pancreatic β cells [54]. In our literature review, we could not find any publication which evaluated the enzyme inhibition of *R. lutea* extracts against α-glycosidase, α-amylase and acetylcholinesterase. This study provides a reference for this for the first time.

#### Cytotoxic activity

**Figure 6:**
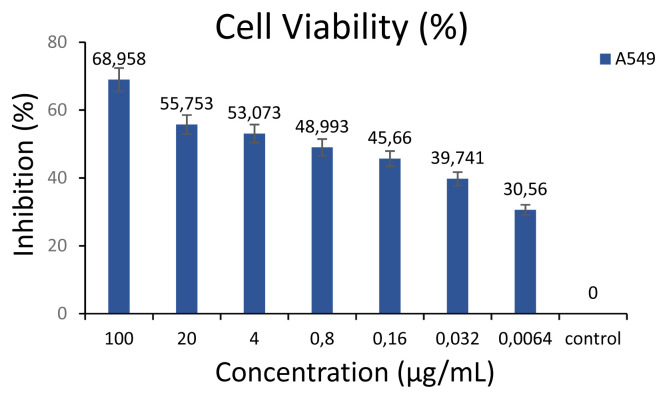
% inhibition values of A549 cells.

Cancer is described as the rapid and uncontrolled growth of certain cells that can multiply in the body and initiate abnormal growth in other areas. These cells can clump together to form a tumor. Cancer is an extremely deadly disease, and, lung cancer is the leading cause of death among cancer types [55]. Most lung cancer patients initially respond to chemotherapy, but later develop resistance to the drug, resulting in cancer recurrence [56]. Chemotherapeutics are used in cancer treatment, but these drugs can cause serious side effects and excessive damage to normal cells. This encouraged researchers to discover new and less toxic anticancer compounds through chemical synthesis or isolation from plants. Many compounds with medicinal plant origin have potential cytotoxicity against various cancer cells [55]. Isolated plant-derived bioactive compounds such as paclitaxel, vinblastine and camptothecin, which are used frequently in recent years, can be used to treat various types of cancer [57]. Kaempferol, isorhamnetin, myricetin, and quercetin flavonoids have anticancer effects on different cancer cell lines through apoptosis [58]. Studies indicated that flavonoid-induced apoptosis may be dependent on the number of hydroxyl groups in the 2-phenyl group and the presence of 3-hydroxyl group [59]. The IC_50_ values of isorhamnetin glycosides purified from *Opuntia Ficus-indica* against NIH 3T3, HT-29 and Caco2 cell lines were determined to range from 8.6 ± 1.8 to 65.9 ± 0.9 μg/mL [58]. In this study, it was demonstrated by the MTT test that EERL caused dose-dependent cytotoxicity in A549 human lung cancer cell lines (p < 0.001) (Table 4). The EERL extract was found to significantly reduce the survival of the A549 cell line with an IC_50_ value of 3.58 ± 1.10 μg/mL (Table 4 and Figure 6). It is thought that this strong cytotoxicity is due to the high amounts of flavonoids and α-glycosides contained in *R. lutea* [18–20]. In a study, treatments of human A375 (melanoma) cell lines for 24 h with increasing concentrations of *R. lutea* autolysates (root, flower and fruits) were examined and the flower of *R. lutea* significantly reduced the survival of the cell line with IC_50_ value of 5.0 ± 0.2 μg/mL [7]. These results appear to agree with the present study and according to our literature review, this study is the first to demonstrate the cytotoxic effect of *R. lutea* on A549 (lung cancer cell line).

### 3.3. Molecular docking studies

Molecular docking studies provide very important information in the drug discovery and evaluation process. With accurate docking scoring functions, rapid and accurate determination of new inhibitors against molecular targets is possible [60]. Based on the results from all these in vitro studies, the binding of isolated compounds with AChE, α-glycosidase and α-amylase enzymes was investigated using molecular docking simulation. Molecular docking analysis was performed using Autodock tools 1.5.7 [36]and BIOVIA Discovery Studio.

**Figure 7:**
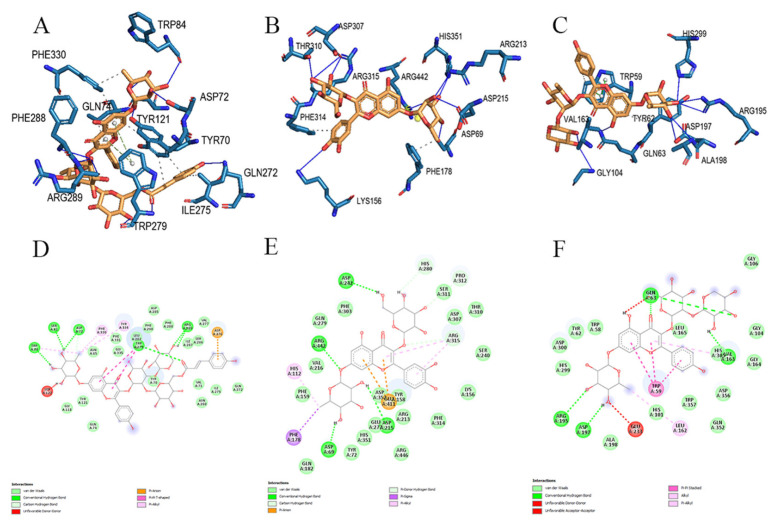
Interactions of AChE (PDB ID 1ACJ) with compound-2; (A) (three-dimensional) and (D) (two-dimensional) represents diagrams. Interactions of α-glycosidase (PDB ID 3A4A) with compound-4a; (B) (three-dimensional) and (E) (two-dimensional) represents diagrams. Interactions of α-amylase (PDB ID 3L2M) with compound-3; (C) (three-dimensional) and (F) (two-dimensional) represents diagrams. Ligands are represented in orange sticks.

Molecular docking analysis was performed to investigate the interactions that occurred during inhibition between isolated compounds and AChE. According to the results, compound-2 had a docking score of −10.8 kcal/mol, the highest score (Table 5). The TRP279 region, which is a hydrophilic pocket in the protein structure, is important in terms of π-π interactions with the A and C rings of the aglycon in compound-2 (Figures 1 and 2) and also 1 H bonding with -(6-p-coumaryl)-β-D glucopyranosyl (R_1_). There was π-anion interaction between the hydrophobic pocket formed by the phenyl ring of R_1_ and the amino acid residue ASP276; 3 hydrogen bonds between amino acid residues TRP84, SER81 and ASP72 and -O-α-L-rhamnopyranosil attached to the aglycone; and 2 hydrogen bonds formed between and ARG289 and -O-β-D-xylopyrosyl (R_2_). In total, there were 11 hydrogen bonds formed between compound-2 and the active site residues ASP72, TRP84, GLN272, TRP279, TRY121, SER122, PHE288 and ARG289 of AChE (Figures 7A and 7D; see also supporting information). These interactions ensured that the ligand binds to the protein structure with strong affinity. Compound-1 had the second highest score with binding energy of −9.3 kcal/mol. According to the molecular docking studies between other ligands and AChE, it was observed that the binding took place in regions other than the active region. Therefore, compounds 3, 4a, 4b, 5a, and 5b did not have any docking score (Table 5). According to the results, only the isolated compounds-1 and 2 were determined to display strong binding with AChE. These docking scores support the in vitro results of this study. Compound-4a exhibited the highest docking score with the α-glycosidase enzyme, calculated as −10.5 kcal/mol (Table 5). Figure 7B represents 3D interactions and 7E represents 2D interactions. There were 2 π-anion interactions between the amino acid residue GLU411 and the A and C rings of the aglycon of compound-4a (Figures 1 and 3) and π-alkyl interactions between the C and B rings of the aglycon with ARG315 residue. The strong H bonds, occurring between the amino acid residues ARG442, ASP215 and ASP69 and the aglycon-bound O-α-L-rhamnopyranosyl (R_1_) as well as ASP242 and the aglycone-bound -O-β-D glucopyranosyl (R_2_), were found to support strong affinity between protein and ligand. Compound-4a formed 9 hydrogen bonds with the active site residues ASP69, LYS156, ARG213, ASP215, ASP307, THR310, ARG315, HIS351 and ARG442 of α-glycosidase (see supporting information). Figure 7C represents 3D interactions and 7F represents 2D interactions from the compound-3 and the α-amylase docking study. The docking score of compound-3 with α-amylase (−10.0 kcal/mol) was the highest score (Table 5). There were 3 π-π stacked interactions between TRP59 in the protein structure and the A, B and C rings of the aglycon of compound-3 (Figures 1 and 3), and hydrogen bonds between GLN 63 and the C ring. It was shown that O- α-L-rhamnopyranosil (R_1_) bound to aglycone interacted hydrophobically with HIS101, and R_1_ also formed 2 hydrogen bonds with ARG195 and ASP197. -O-xylopyrosyl-α-L-rhamnopyranosyl (R_2_) bound to aglycone formed an H bond with VAL163. In total, there were 11 hydrogen bonds formed with the active site residues GLN 63, GLY104, VAL163, ARG195, ASP197, ALA198 and HIS 299 between compound-3, which supported the binding of the ligand to the protein with a strong affinity (Figures 7C and 7F; see also supporting information). All 7 compounds were determined to bind strongly with α-glycosidase and α-amylase (Table 5). The results supported the strong interaction between protein and ligand structures. In this study, the high docking score for α-glycosidase and α-amylase enzymes with 7 kaempferol and isorhamnetin molecules isolated from *R. lutea* supported the in vitro results showing that *R. lutea* significantly inhibited these antidiabetic enzymes.

As a result, this study provides very important information about the phytochemical properties and bioactivity of *Reseda lutea* L. regarding antioxidant activity, phenolic and flavonoid contents and inhibition potential for some enzymes related to the treatment of Alzheimer’s disease and diabetes mellitus, and cytotoxic activity. It was determined EERL and WERL plant extracts had powerful antioxidant potential. Moreover, EERL showed very high inhibitory effect against AChE, α-amylase and α-glycosidase enzymes. Also, the highly cytotoxic effect of EERL, which is thought to be caused by *R. lutea*’s high flavonoid content detected in vitro, was observed on human lung cancer cell lines (A549). In silico studies were included in order to determine the effects of isolated flavonoid compounds on Alzheimer’s disease and diabetes mellitus mechanisms and to support the results of in vitro experiments. The affinities of 7 kaempferol and isorhamnetin rhamnopyranosides molecules isolated from *R. lutea* to AChE, α-amylase and α-glycosidase were determined by molecular docking studies. Molecular docking studies demonstrated that strong anticholinergic and antidiabetic activity of *R. lutea* was associated with the ability of its flavonoid content to inhibit AChE, α-amylase and α-glycosidase enzymes. In vitro and in silico studies support the potential pharmacological activity of *R. lutea* for drug design processes related to the treatment of Alzheimer’s disease, diabetes mellitus and lung cancer. This study is also a preliminary study in terms of performing in vivo experiments.

## Supplementary material

**Table:** The parameters of the H-bond interactions between the for 7 phenolic compounds isolated from R. lutea and enzymes.

*R. lute*a	AChE (PDB ID 1ACJ)		α-glycosidase (PDB ID:3A4A)		α-amylase (PDB ID: 3L2M)	
H-bond	Residues	Bond length (A)	Residues	Bond length (A)	Residues	Bond length (A)
Compound-1	Asp72	2,89	Gln279	3,01	Trp59	2,75
	Arg289	2,37	His280	2,89	Ala198	2,98
	Gly335	3,31	Ala281	2,95	Lys200	2,19
	Try121	2,61	Asn302	2,07	His201	2,89
	Ser122	1,84	Ser304	1,96	Glu233	3,42
	Phe288	3,28	Asp307	2,64	His299	3,3
			Gly309	2,1	His305	2,84
			Thr310	3,26; 2,95	Asp356	1,91
			Arg315	2,44; 3,29		
			Asp352	3,29		
			Arg442	3,08		
Compound-2	Asp72	2,93	Gln279	2,7	Trp59	2,4
	Trp84	2,25	His280	1,73	Tyr151	3,19
	Gln272	2,85	Ala281	2,19; 2,67	Ala198	3,31
	Trp279	2,28; 3,14	Asn302	2,14	Lys200	3,18
	Try121	2,35	Ser304	2,78	His201	2,75
	Ser122	1,79; 1,81	Ser304	2,78	Glu233	2,67
	Phe288	3,14	Arg315	2,78; 2,82	Glu240	2,77
	Arg289	2,46; 2,10	Asp352	2,23	Asp300	2,11
			Glu411	3,25		
Compound-3			Asp69	1,53	Gln63	2,45
			Lys156	2,71; 3,29	Gly104	3,29
			Arg213	2,7	Val163	2,38
			Ser241	3,08	Arg195	2,77; 2,55
			Asp242	1,97	Asp197	2,8
			Pro312	2,64; 2,77	Ala198	3,28
			Arg315	2,88	His299	2,82
			His351	1,84		
			Asp352	2,55		
			Arg442	3,19		
Compound-4A			Asp69	1,66	Trp59	2,4
			Lys156	3,37	Gln63	2,57
			Arg213	2,51	Arg195	2,6
			Asp215	2,28	Ala198	3,6
			Asp307	3,48	His201	2,33
			Thr310	4,03	Glu233	2,60; 2,62
			Arg315	2,16	Asp300	2,29
			His351	1,82	His305	2,47; 2,20
			Arg442	3.59		
Compound-4B			His280	2,04	Gln63	2,41
			Asp307	2,56	Val163	2,29
			Thr310	2,02	Arg195	3,45; 2,28
			Arg315	3,34	Ala198	3,48
			Glu411	2,87	His201	2,76
					Glu233	2,72; 2,19
					His299	2,45
					His305	2,39
Compound-5A			Lys156	2,43	Arg195	3,18; 2,20
			Thr310	3,57; 3,58	Lys200	3,49
			Glu411	2,55; 1,90	His201	2
					Glu233	2,22
					His299	1,82
Compound-5B			Ser157	2,55	Arg195	2,89; 2,01
			Asp242	3,31	Asp197	2,32
			Asp307	2,39; 3,26	Ala198	3,67
			Gly309	2,96	Lys200	3,36
			Pro312	2,69	His201	2,26
					His299	1,74

## Figures and Tables

**Table 1:** The reducing power of EERL, WERL and standard antioxidant compounds as well as IC_50_ (μg/mL) values of DPPH^•^, Fe^+2^ chelating, ABTS^•^^+^ scavenging activities.

Antioxidants	Fe^3+−^Fe^2+^ reducing*		Cu^2+^-Cu^+^ reducing*		Fe^3+^-TPTZ reducing*		DPPH^•^ scavenging		ABTS^•+^ scavenging		Fe^+2^ chelating	
	λ_700_	r^2^	λ _450_	r^2^	λ _593_	r^2^	IC_50_	r^2^	IC_50_	r^2^	IC_50_	r^2^
**Asc. Acid (a)**	1.52 ± 0.03^c,b,d,e^	1.00	1.07 ± 0.01^c, d,e^	0.97	1.62 ± 0.02^c,b,d,e^	0.99	16.12 ± 0.01	0.96	23.10 ± 0.01	1.00	99.0 ± 0.04	1.00
**BHT(b)**	1.27 ± 0.01^c,d,e^	0.99	1.56 ± 0.09^c,d,e^	1.00	0.91 ± 0.01 ^c,d,e^	0.99	31.50 ± 0.01	0.98	26.65 ± 0.01	0.97	14.75 ± 0.06	0.97
**α-tocop.** **(c)**	0.99 ± 0.01^e^	0.99	0.79 ± 0.07^e^	1.00	0.76 ± 0.08^d,e^	0.99	23.10 ± 0.03	0.98	15.40 ± 0.01	0.99	330.0 ± 0.02	0.91
**EERL(d)**	1.00 ± 0.01^c,e^	0.99	0.85 ± 0.01^c,e^	0.99	0.64 ± 0.03d	0.99	231.0 ± 0.01	0.95	23.90 ± 0.01	0.97	11.18 ± 0.05	0.96
**WERL(e)**	0.08 ± 0.01	0.97	0.11 ± 0.01	1.00	0.35 ± 0.01^e^	0.96	346.50 ± 0.03	0.96	14.14 ± 0.04	0.99	-	-
**EDTA(f)**	-	-	-	-	-	-	-	-	-	-	231.0 ± 0.30	0.95

* Different letters in the same column indicate statistically significant difference between the means (p < 0.001 regarded as significant).

**Table 2:** The total phenolic and flavonoid contents of WERL and EERL.

Extracts	Total phenolics (μg/mg extract)	Total flavonoids (μg/mg extract)
**EERL**	47.73 ± 0.32	84.43 ± 2.72
**WERL**	7.73 ± 0.13	9.83 ± 0.41

**Table 3:** The enzyme inhibition (IC_50_, μg/mL) of EERL against α-glycosidase, α-amylase and AChE.

Enzymes	EERL		Standards
	IC_50_	r^2^	IC_50_
**α-Glycosidase** a	1.38	0.97	22.80
**α-Amylase** a	0.11	0.97	10.01
**AChE** b	2.21	0.99	0.124

a Acarbose was used as positive control for α-glycosidase and α-amylase enzymes and taken from reference of [44], [45], respectively.

b Tacrine was used as positive control for AChE enzyme and taken from reference of [46].

**Table 4:** Descriptive statistics and comparisons of MTT tests.

Cell lines	Concentrations (μg/mL)	n	Mean ± SD	p
**A549**	100 (a)	5	0.98 ± 0.05	0.001
	20 (b)	5	1.40 ± 0.03 ^a^	
	4 (c)	5	1.48 ± 0.04 ^a,b^	
	0.8 (d)	5	1.71 ± 0.05 ^a,b,c^	
	0.16 (e)	5	1.72 ± 0.03 ^a,b,c,d^	
	0.032 (f)	5	1.90 ± 0.02 ^a,b,c,d,e,g^	
	0.064 (g)	5	2.19 ±0.02 ^a,b,c,d,e^	
	0 (Control) (h)	5	3.15 ± 0.08 ^a,b,c,d,e,f,g^	

Different letters in the same column indicate statistically significant difference between the means.

**Table 5:** The docking scores of isolated compounds from *R. lutea*.

No	Compounds	Docking scores		
		AChE	α-glycosidase	α-amylase
**1**	Compound-1	−9.3	−9.5	−9.1
**2**	Compound-2	**−10.8**	−10.4	−9.6
**3**	Compound-3	-	−10.4	**−10.0**
**4**	Compound-4a	-	**−10.5**	−9.2
**5**	Compound-4b	-	−9.5	−9.3
**6**	Compound-5a	-	−9.5	−9.8
**7**	Compound-5b	-	−9.5	−9.4
